# Electronic Health Record–Based Prediction of 1-Year Risk of Incident Cardiac Dysrhythmia: Prospective Case-Finding Algorithm Development and Validation Study

**DOI:** 10.2196/23606

**Published:** 2021-02-17

**Authors:** Yaqi Zhang, Yongxia Han, Peng Gao, Yifu Mo, Shiying Hao, Jia Huang, Fangfan Ye, Zhen Li, Le Zheng, Xiaoming Yao, Zhen Li, Xiaodong Li, Xiaofang Wang, Chao-Jung Huang, Bo Jin, Yani Zhang, Gabriel Yang, Shaun T Alfreds, Laura Kanov, Karl G Sylvester, Eric Widen, Licheng Li, Xuefeng Ling

**Affiliations:** 1 School of Electrical Power Engineering South China University of Technology Guangzhou China; 2 Department of Surgery Stanford University Stanford, CA United States; 3 College of Pharmacy Shandong University of Traditional Chinese Medicine Shandong China; 4 China Southern Power Grid Company Limited Guangzhou China; 5 Department of Cardiothoracic Surgery Stanford University Stanford, CA United States; 6 Clinical and Translational Research Program Betty Irene Moore Children's Heart Center Lucile Packard Children’s Hospital Palo Alto, CA United States; 7 Department of Critical Care Medicine The Third People’s Hospital of Shenzhen The Second Affiliated Hospital of Southern University of Science and Technology Shenzhen China; 8 Department of Anesthesiology The Third People’s Hospital of Shenzhen The Second Affiliated Hospital of Southern University of Science and Technology Shenzhen China; 9 Translational Medicine Laboratory Queen Mary Hospital Hong Kong University Hong Kong China; 10 School of Electrical Engineering Southeast University Nanjing China; 11 School of Computer Science and Technology Hangzhou Dianzi University Hangzhou China; 12 Shandong Provincial Key Laboratory of Network Based Intelligent Computing University of Jinan Jinan China; 13 National Taiwan University–Stanford Joint Program Office of Artificial Intelligence in Biotechnology Ministry of Science and Technology Joint Research Center for Artificial Intelligence Technology and All Vista Healthcare Taipei China; 14 HBI Solutions Inc Palo Alto, CA United States; 15 Tianjin Yunjian Medical Laboratory Institute Co Ltd Tianjing China; 16 The Harker School San Jose, CA United States; 17 HealthInfoNet Portland, ME United States

**Keywords:** cardiac dysrhythmia, prospective case finding, risk stratification, electronic health records

## Abstract

**Background:**

Cardiac dysrhythmia is currently an extremely common disease. Severe arrhythmias often cause a series of complications, including congestive heart failure, fainting or syncope, stroke, and sudden death.

**Objective:**

The aim of this study was to predict incident arrhythmia prospectively within a 1-year period to provide early warning of impending arrhythmia.

**Methods:**

Retrospective (1,033,856 individuals enrolled between October 1, 2016, and October 1, 2017) and prospective (1,040,767 individuals enrolled between October 1, 2017, and October 1, 2018) cohorts were constructed from integrated electronic health records in Maine, United States. An ensemble learning workflow was built through multiple machine learning algorithms. Differentiating features, including acute and chronic diseases, procedures, health status, laboratory tests, prescriptions, clinical utilization indicators, and socioeconomic determinants, were compiled for incident arrhythmia assessment. The predictive model was retrospectively trained and calibrated using an isotonic regression method and was prospectively validated. Model performance was evaluated using the area under the receiver operating characteristic curve (AUROC).

**Results:**

The cardiac dysrhythmia case-finding algorithm (retrospective: AUROC 0.854; prospective: AUROC 0.827) stratified the population into 5 risk groups: 53.35% (555,233/1,040,767), 44.83% (466,594/1,040,767), 1.76% (18,290/1,040,767), 0.06% (623/1,040,767), and 0.003% (27/1,040,767) were in the very low-risk, low-risk, medium-risk, high-risk, and very high-risk groups, respectively; 51.85% (14/27) patients in the very high-risk subgroup were confirmed to have incident cardiac dysrhythmia within the subsequent 1 year.

**Conclusions:**

Our case-finding algorithm is promising for prospectively predicting 1-year incident cardiac dysrhythmias in a general population, and we believe that our case-finding algorithm can serve as an early warning system to allow statewide population-level screening and surveillance to improve cardiac dysrhythmia care.

## Introduction

Cardiac dysrhythmia is a series of conditions in which the heartbeat is irregular, too fast, or too slow. There are many types of dysrhythmias, and most are mild; however, some severe arrhythmias increase the risk of serious or even life-threatening complications, such as congestive heart failure, syncope, stroke, and sudden death. More than 850,000 people are hospitalized for arrhythmias each year in the United States [1]. Sudden cardiac death is the cause of approximately half of the deaths due to cardiovascular disease and approximately 15% of all deaths globally [2]. Approximately 80% of sudden cardiac deaths are caused by ventricular arrhythmias [3]. If the risk and severity of cardiac arrhythmias can be accurately predicted, actionable medical treatments can be applied to proactively reduce incidence and prevent disease deterioration.

A few arrhythmia risk prediction tools have been applied in programs for screening, prevention of life-threatening arrhythmias [4], and selection of therapy and intervention [5]. Most models were developed for specific populations or special conditions: a prediction model was developed in a consecutive cohort of 1138 patients who underwent carotid endarterectomy [6] with a C statistic of 0.69 (0.64-0.73); a ^123^I-metaiodobenzylguanidine single photon emission computed tomography model had an area under the receiver operating characteristic curve (AUROC) of 0.76 [7]; and a cardiac magnetic resonance imaging with late gadolinium enhancement model, with AUROC values ranging from 0.721 to 0.812 [8] for different scar characteristics, relied on specially captured information that was not available for risk prediction in a large-population cohort. The main limitations of these models included small sample sizes from a single source of data, lack of consideration of the interactions among multiple risk factors, and insufficient real-time monitoring of predictor changes [6,9]. To date, no well-performing and widely recognized risk assessment model has been implemented for clinical application in a large general population.

With the widespread use of electronic health records (EHRs) in hospitals and clinics, an individual's physical and mental condition can be assessed to potentially improve the effectiveness of health management [10]. Individuals' comprehensive clinical histories have been used to build risk models with various risk factors [11]. The multidimensional clinical data elements and the generality of the EHR-based data sets are promising to extract more comprehensive risk patterns [12]. Empowered by statewide health information exchange platforms, we applied advanced machine learning including deep learning analytics to deliver actionable information that can help health care organizations identify high-risk individuals, which could improve patients’ health and lower costs [13,14].

The purpose of this study was to retrospectively develop and prospectively validate our case-finding algorithm for patients at risk of 1-year incident cardiac dysrhythmia in Maine, United States.

## Methods

### Ethics Statement

Protected personal health information was deidentified for model development. Due to the nature of the development with deidentified data, this study was exempted from ethics review by the Stanford University Institutional Review Board (October 16, 2017).

### Experimental Design and Workflow

**Figure 1:**
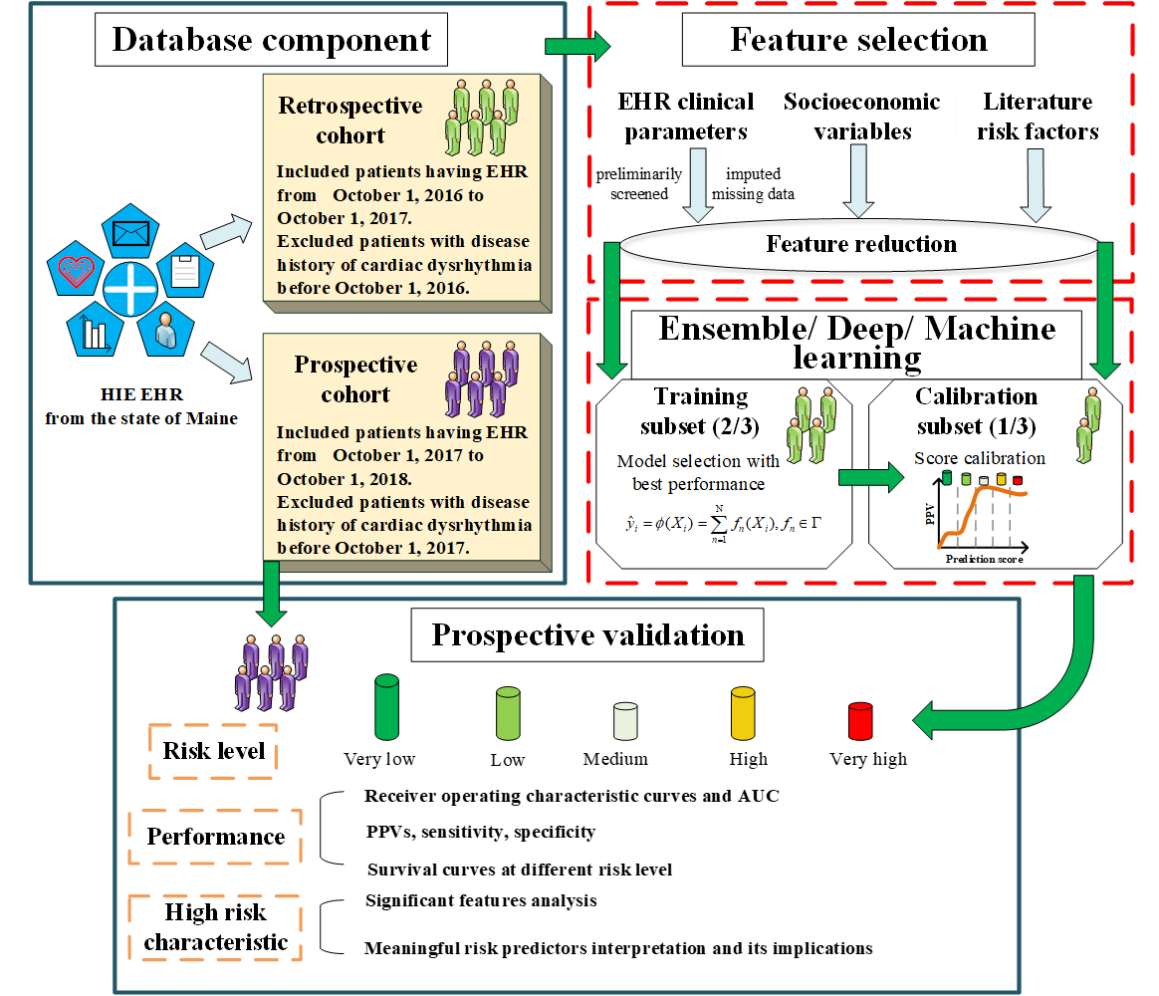
A workflow diagram that describes data collection, data classification, model building, and model evaluation. AUC: area under the curve; EHR: electronic health record; HIE: health information exchange; PPV: positive predictive value.

**Figure 2:**
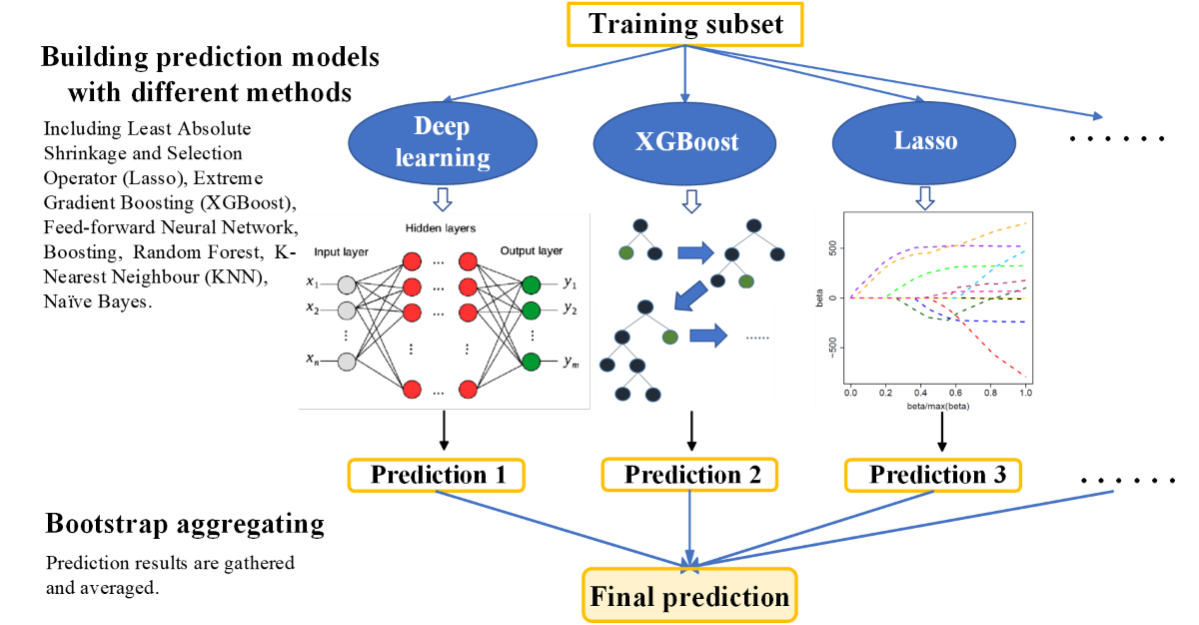
The detailed modeling process with the ensemble learning method.

A complete workflow (data collection, exclusion, and application) is presented in Figure 1. Figure 2 illustrates the detailed modeling process with an ensemble learning method.

### Data Sources

Nearly 95% of the population in Maine was included in the study. Clinical variables were collected, including demographic information, socioeconomic status, laboratory, and radiographic tests coded according to Logical Observation Identifier Names and Codes, outpatient medication prescriptions coded according to the National Drug Code, and primary and secondary diagnoses and procedures, which were coded using International Classification of Diseases, Tenth Revision, Clinical Modification (ICD-10-CM).

### Definition of Cardiac Dysrhythmia

Cardiac dysrhythmia was defined according to ICD-10-CM, including paroxysmal supraventricular tachycardia, paroxysmal ventricular tachycardia, atrial fibrillation, atrial flutter, premature beats, sinoatrial node dysfunction, and other cardiac dysrhythmias (ICD-10-CM diagnosis codes from I47 to I49).

### Study Population

The individuals included in this study were patients who visited any medical institutions in the Maine health information exchange network from October 1, 2015, to October 1, 2018. The retrospective timeframe was from October 1, 2016, to October 1, 2017. The prospective timeframe was from October 1, 2017, to October 1, 2018. Individuals were excluded if they died during the study period or were diagnosed with cardiac dysrhythmia before October 1, 2016, for the retrospective analysis and before October 1, 2017, for the prospective analysis.

### Features

Information regarding the medical history, diagnoses, medications, treatment plans, immunization dates, allergies, radiology images, and laboratory test results were extracted from EHRs. Relevant socioeconomic variables were extracted from the US Census and US Department of Agriculture websites [15,16] (see Multimedia Appendix 1). Individuals were aggregated into several age categories. Socioeconomic age-related features such as work or retirement status and conditions about insurance and pension status were structured and standardized as socioeconomic features.

Missing values in the data matrix for machine learning most likely arise due to the lack of the order of the tests or lack of coding for the absence of relevant comorbidity. The data matrix was constructed with all the entries to document the binary outcome (0 or 1) or the counts of the utilization. Therefore, missing entries caused by the data matrix consolidation were filled as 0 outcome or 0 count. Given the confounding effects of stratification factors among various features in the large number of samples, the Cochran-Mantel-Haenszel test was applied to analyze the relationship between the features and corresponding outcome under age-group strata [17]. A total of 658 features were screened out of the original 17,865 features for the subsequent modeling analytics.

Multihypothesis test correction was conducted to ensure the false discovery rate of the remaining features was in an acceptable range [18].

### Correlation Networks

To investigate associations between feature categories, we built correlation networks among the features based on Spearman correlations. In these networks, vertices correspond to features, and an edge existed between 2 vertices if and only if a correlation (absolute value of the Spearman coefficient) >0.1 between 2 features was observed. In real clinical settings, these features are most likely not independent of each other, and more complicated causative or associative relationships may exist among these significant feature categories.

### Model Building

The retrospective cohort was divided into 2 parts: two-thirds of the data in the retrospective cohort were used for training, and the remaining one-third was used for model calibration. For training, multiple algorithms were applied, including least absolute shrinkage and selection operator (LASSO) [19], feed-forward neural network [20], random forest [21], boosting [22], extreme gradient boosting (XGBoost) [23,24], naïve Bayes [25], and k-nearest neighbor [26]. Bayesian probabilistic ensemble setting was adopted to integrate various model results for better performance [27]. The hypothesis defines a conditional probability distribution of

*h*(*x*) = *P*(*f*(*x*) = *y* | *x*, *h*) (**1**)

where *x* is a given data point. The ensemble method in which the ensemble consists of all of the hypotheses in *H* each weighted by its posterior probability *P*(*h* | *x*), which we used as the positive predictive value (PPV) for each individual model, can be expressed as:



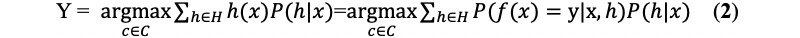



in which each hypothesis is also multiplied by the prior probability of that hypothesis and where *Y* is the integrated predicted class, *C* is the set of all possible classes in the training space predicted label, and *c* indicates a specific class in each classifier *f*(*x*) = *y* | *x*.

The isotonic regression method was used to calibrate the model [28], producing a calibrated value *y'*. The *y'* estimates of the calibration subset were calculated and mapped to PPVs. The PPV for a certain *y'* was the corresponding proportion of incident arrhythmia events in the cohort having predictive estimates equal to or larger than this *y'*. Therefore, our risk scores, quantifying the probability of an incident arrhythmia event within the subsequent 1 year, can be interpreted by the PPVs.

### Model Evaluations

To find individuals at different risk levels, 5 risk groups were created and assigned to bins: very low-risk, low-risk, medium-risk, high-risk, and very high-risk bins. The model performance was evaluated through sensitivity, specificity, and PPV. The AUROC values were utilized to illustrate the relationship between sensitivity and specificity by composition.

Survival analysis was applied to track the timing of arrhythmia diagnosis in different risk bins. Kaplan-Meier curves were plotted for different risk levels to stratify the time to events of new incidences. The Cox proportional hazards regression method was used for multivariate analysis.

## Results

### Baseline Characteristics

The cohort baseline characteristics are shown in Table 1. There was no obvious difference in demographic and clinical patterns between the retrospective and prospective cohorts.

**Table 1:** Baseline characteristics of the retrospective and prospective cohorts.

Characteristic			Retrospective (n=1,033,856), n (%)		Prospective (n=1,040,767), n (%)
**Age (years)**					
	<35	391,613 (37.90)		399,545 (38.40)	
	35-50	178,348 (17.20)		176,995 (17.00)	
	50-65	245,580 (23.80)		243,161 (23.40)	
	65-75	132,444 (12.80)		135,600 (13.00)	
	>75	85,871 (8.30)		85,466 (8.20)	
**Gender**					
	Male	464,796 (45.00)		571,821 (54.90)	
	Female	569,060 (55.00)		468,946 (45.10)	
**Chronic disease**					
	Cardiovascular disease^a^	215,059 (20.80)		228,757 (22.00)	
	Chronic obstructive pulmonary disease	38,029 (3.70)		42,778 (4.10)	
	Chronic kidney disease	21,277 (2.70)		23,653 (2.30)	
	Type 2 diabetes	83,387 (8.10)		84,649 (8.10)	
	Disorder of metabolism	214,606 (20.80)		221,168 (21.30)	
	Hypothyroidism	73,319 (7.10)		74,495 (7.20)	
**Acute disease**					
	Pain in throat and chest	48,507 (4.69)		55,368 (5.30)	
	Anemia	10,921 (1.06)		11,099 (1.10)	
	Edema	17,096 (1.65)		17,768 (1.70)	
	Syncope and collapse	10,662 (1.03)		11,855 (1.10)	
	Malaise and fatigue	47,793 (4.62)		46,834 (4.50)	
**Health status**					
	Long term (current) drug therapy	86,501 (8.40)		107,834 (10.40)	
	Personal history of other diseases and conditions	104,282 (10.10)		130,955 (12.60)	
	BMI>33.0	3055 (0.30)		3842 (0.40)	
**Laboratory test**					
	Glomerular filtration rate	391,613 (37.90)		16,726 (1.60)	
	Coagulation assay	12,197 (1.20)		9616 (0.90)	
	Carboxyhemoglobin in blood	35,773 (3.50)		20,850 (2.00)	
**Medication**					
	Beta-adrenergic blocker	66,750 (6.50)		69,572 (6.70)	
	Proton pump inhibitor	68,123 (6.60)		69,278 (6.70)	
	Vitamin K antagonist	6858 (0.70)		5541 (0.50)	

^a^Cardiovascular diseases included heart failure, rheumatic mitral valve diseases, atrioventricular and left bundle-branch block, cardiomyopathy, nonrheumatic aortic, tricuspid and mitral valve disorders, atherosclerosis, and other disorders of arteries and arterioles.

### Feature Community Structure and Correlation Networks

The original features (n=17,865) were extracted from the EHRs and socioeconomic databases. The model building process identified 307 features with contributing weights, including 2 demographic features, 18 socioeconomic characteristics, 101 chronic disease diagnostics, 147 confirmed acute disease and disease events, 7 procedures, 5 utilization variables, 9 factors of the health status, 9 medication prescriptions, and 9 laboratory tests. The top 60 important features and their odds ratios in the model are tabulated in Multimedia Appendix 2.

**Figure 3:**
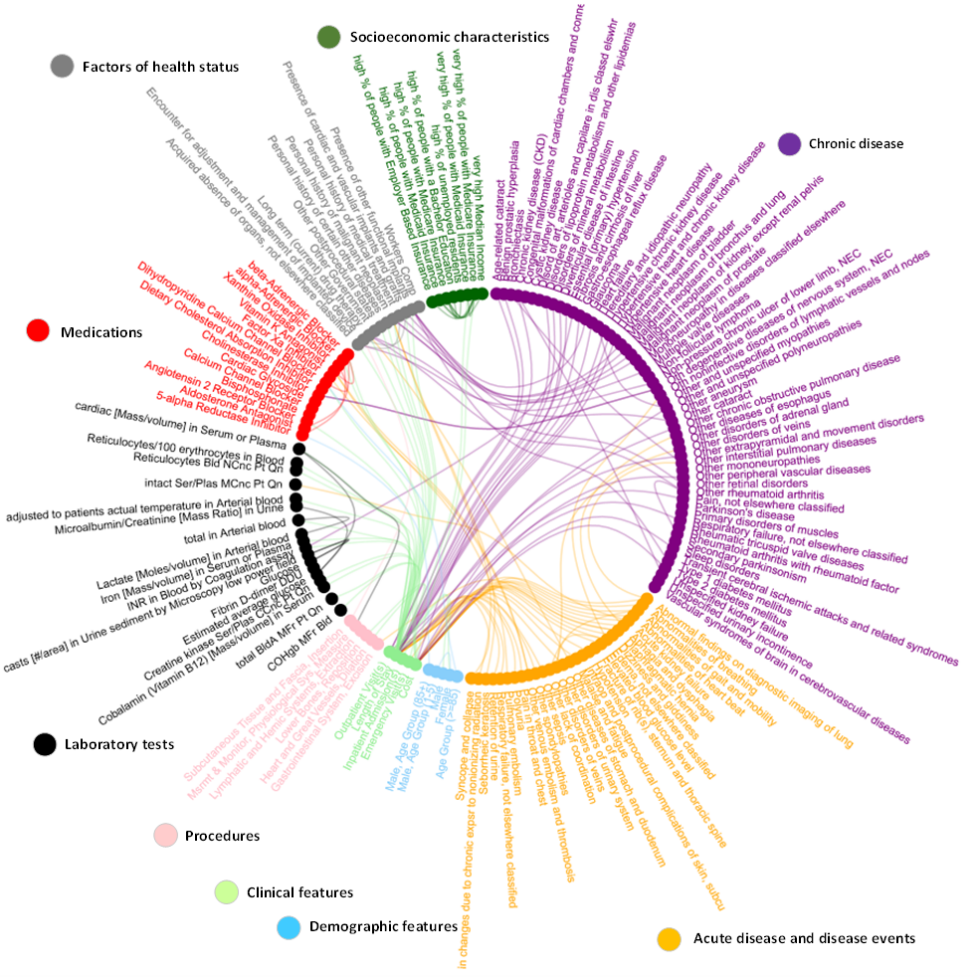
The community structure of the 153 impactful features and their correlation networks (absolute value of the correlation coefficient >0.1).

**Figure 4:**
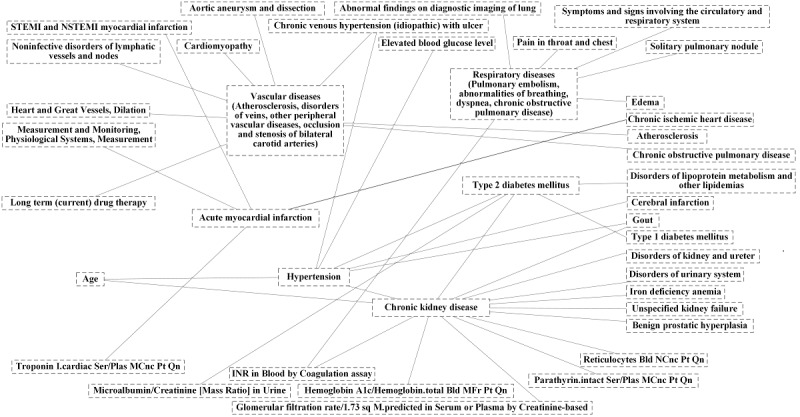
The important network structure of the predictive diagnostic features.

We built correlation networks among these 307 features based on Spearman correlations. The integral correlation networks contain 307 vertices and 325 edges. The majority of edges involved diagnostic diseases (n=206) and demographic features (n=34), with an additional 28 edges involving clinical medications, 27 involving laboratory tests, 18 involving health status, 15 involving procedures, and 10 involving socioeconomic characteristics, as well as all the utilization variables. The community structure of the 153 impactful features in different types and their correlation networks containing 127 edges, as an example, is shown in Figure 3. The important network structure of the predictive diagnostic features is shown in Figure 4.

Arrhythmia is an important group of cardiovascular diseases and is associated with other cardiovascular diseases. Heart disease–related features were revealed in our community structures and correlational networks, including acute myocardial infarction, aortic dissection, chronic ischemic heart disease, cardiomyopathy, and atherosclerosis caused by chronic obstructive pulmonary disease. These features imply a potential causative relationship with arrhythmia. Electrolyte imbalance, heart enlargement, heart failure, and myocardial ischemia may be related to the pathogenesis of arrhythmia and may be complications of chronic kidney disease, metabolic syndrome (type 2 diabetes), and hypertension. The associative relationship between arrhythmia and these chronic diseases is shown in Figure 4. Chronic kidney disease patients in later stages can have comorbidity with oliguria, anuria, or uremic cardiomyopathy, leading to uremic toxin accumulation in the body, and imbalance of homeostasis, leading to arrhythmia attack. Diabetes can cause microvascular and macrovascular complications with different pathological mechanisms, such as diabetic cardiomyopathy and diabetic kidney disease. Hypertension and chronic kidney disease are closely interlinked pathophysiologic states. Anemia is common in patients with heart disease. It was present in approximately one-third of patients with congestive heart failure and 10% to 20% of patients with coronary heart disease. These diseases can lead to cardiac dysfunction, inducing malignant arrhythmia.

### Model Performance

#### AUROC

The AUROC results from our predictive methods, including LASSO, feed-forward neural network, random forest, boosting, XGBoost, naïve Bayes, k-nearest neighbor, and ensemble learning, were compared to demonstrate the effectiveness of the cardiac dysrhythmia risk prediction (Table 2 and Multimedia Appendix 3). An ensemble learning method was applied to harmonize and vote (the results of multiple algorithms) for the best modeling method in this study (Multimedia Appendix 4; AUROC 0.827). In addition, the prediction AUROC (0.819) calculated by LASSO was comparable to that calculated by the ensemble learning method. The following analysis was based on LASSO model predictions.

**Table 2:** Model performance.

Model	AUROC^a^ (95% CI)
Ensemble learning	0.827 (0.824-0.830)
Least absolute shrinkage and selection operator	0.819 (0.816-0.822)
Extreme gradient boosting	0.808 (0.805-0.811)
Feed-forward neural network	0.807 (0.804-0.810)
Boosting	0.775 (0.771-0.778)
Random forest	0.695 (0.691-0.699)
*k*-Nearest neighbor	0.631 (0.627-0.635)
Naïve Bayes	0.611 (0.607-0.614)

^a^AUROC: area under the receiver operating characteristic curve.

#### The Risk Score Metric

**Figure 5:**
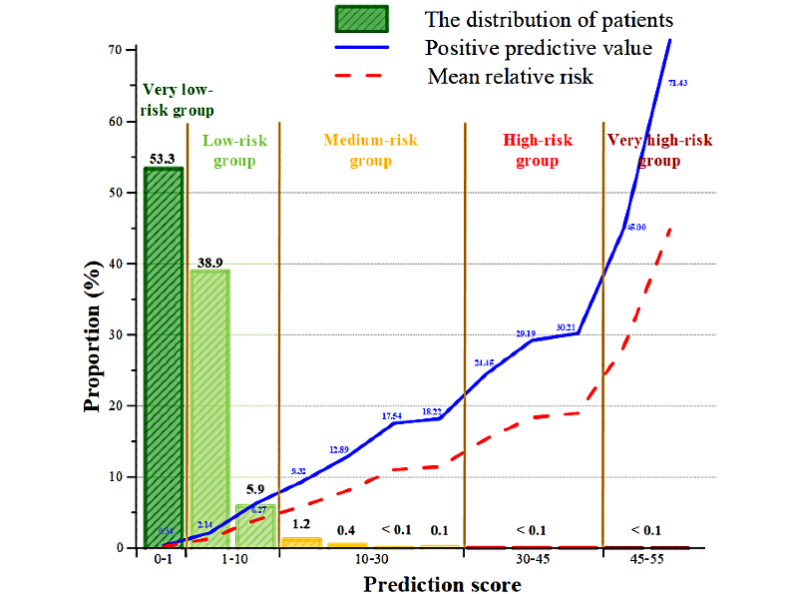
The proportion of patients and their positive predictive values with different prediction scores on the prospective cohort.

Patients in the prospective cohort were divided into 5 risk categories (very low, low, medium, high, and very high; Figure 5) based on the predictive scores. Over 90% of the prospective patients (1,021,827/1,040,767) were categorized into the very low-risk or low-risk categories, while 0.063% (650/1,040,767) were classified as high-risk or very high-risk; 51.85% of patients (14/27) in the very high-risk group had confirmative diagnosis of arrhythmia in the subsequent year (Multimedia Appendix 5).

**Figure 6:**
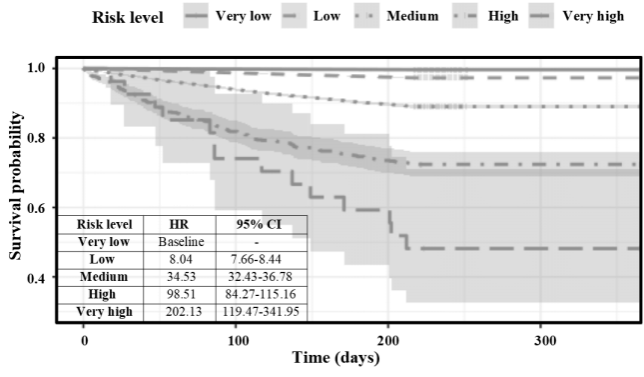
Survival curves of the 5 risk groups. HR: hazard ratio.

Survival curve analysis was applied to quantify the effectiveness of the 5-risk bin stratification for future cardiac dysrhythmia events within 1 year (Figure 6). Survival curves with different risk levels were distinguished with hazard ratios varying from 8.04 to 202.13 at different risk bins. Sensitivities, specificities, and PPVs in the 5 risk groups were documented (Multimedia Appendix 5); 0.34% (1,873/555,233) and 51.85% (14/27) of the patients were stratified as very low-risk and very high-risk, respectively, for arrhythmia in the subsequent 1 year.

### Characterization of the Predictive Clinical Parameters

Age distributions at 5 different risk levels are shown in Multimedia Appendix 6. Young individuals (<35 years) were enriched in the low-risk or very low-risk bins, while older individuals were enriched in the high-risk or very high-risk bins; 68.46% of patients (380,112/555,233) in the very low-risk group were young individuals. The majority of the patients (397,164/466,594, 85.12%) in the low-risk group were older than 50 years. In the medium-, high-, and very high-risk groups, patients aged 65 years or older were the majority (medium: 16,194/18,290, 88.54%; high: 576/623, 92.46%; very high: 27/27, 100%; see Multimedia Appendix 7). There were more male patients than female patients in the medium-risk (10,308/18,290, 56.36%), high-risk (381/623, 61.16%), and very high-risk groups (14/27, 51.85%).

Many patients in the high-risk and the very high-risk groups had comorbidity with chronic (cardiovascular disease: 436/650, 67.07%; metabolism disorder: 304/650, 46.77%; type 2 diabetes: 183/650, 28.15%; chronic obstructive pulmonary disease: 159/650, 24.46%; chronic kidney disease: 146/650, 22.46%) or acute diseases (cardiovascular disease: 220/650, 33.85%; syncope and collapse: 28/650, 4.31%; dizziness and giddiness: 47/650, 7.23%; pain in throat and chest: 81/650, 12.46%; breathing abnormalities: 151/650, 23.23%; Multimedia Appendix 7). Time-to-event analysis was applied to these disease groups (Multimedia Appendix 8). There were apparent differences in time-to-event patterns between patients with low risk and those with high risk. Furthermore, in the high-risk and very high-risk bins, different disease groups demonstrated different patterns. Patients with construction disorders had less probability of experiencing arrhythmia than those with other chronic diseases. Patients with dizziness and giddiness had higher chance of experiencing arrhythmia in a certain time period than those with other acute diseases.

Multimedia Appendix 9 shows the 9 health status features used in the model: (1) long-term (current) drug therapy, (2) personal history of certain other diseases, (3) personal history of other diseases and conditions, (4) BMI >33 kg/m^2^ (overweight), (5) encounter for supervision of normal pregnancy, (6) presence of cardiac and vascular implant and graft, (7) acquired absence of organs, (8) encounter for follow-up examination after completed treatment for conditions other than malignant neoplasm, and (9) presence of other functional implants; 57.85% of patients (376/650) in the high-risk and very high-risk bins had at least 1 abnormal health status feature, in contrast to 23.35% (238,612/1,021,827) in the low-risk and very low-risk bins.

Multimedia Appendix 9 summarizes the 9 laboratory test predictors used in the model: (1) coagulation assay, (2) glomerular filtration rate, (3) carboxyhemoglobin in blood, (4) cardiac troponin T antibodies in blood, (5) blood glucose, (6) creatine kinase, (7) reticulocytes in blood, (8) n-terminal prohormone B-type natriuretic peptide in serum or plasma, and (9) estimated average glucose level. Having abnormal results of the coagulation assay had the highest weight in the model, and 34.62% of patients (225/650) in the high-risk and very high-risk bins had at least 1 abnormal result among the 9 laboratory tests, in contrast to only 5.81% (59,367/1,021,827) in the low-risk and very low-risk bins.

Multimedia Appendix 9 also shows the 9 prescriptions used as predictors in the model: (1) beta-adrenergic blocker, (2) 3-hydroxy-3-methylglutaryl coenzyme A reductase inhibitor, (3) loop diuretic, (4) calcium channel blocker, (5) proton pump inhibitor, (6) vitamin K antagonist, (7) dihydropyridine calcium channel blocker, (8) angiotensin-converting enzyme inhibitor, and (9) factor Xa inhibitor. All drugs, except proton pump inhibitors, were mainly used to treat cardiovascular diseases such as hypertension, congestive heart failure, coronary artery disease, hypertrophic cardiomyopathy, deep vein thrombosis, and acute pulmonary embolism; 90% of individuals (585/650) in the high-risk and very high-risk bins had medication histories with at least 1 drug, in contrast to 16.67% (170,317/1,021,827) of individuals in the low-risk and very low-risk bins.

Socioeconomic features reflected the social disparities of individuals’ living environments and living conditions. The Spearman rank method was used to study the correlation between socioeconomic factors and arrhythmia (Multimedia Appendix 10). Our analysis revealed a high enrichment of higher education (ρ=–0.0036), high-income families (ρ=–0.0218), and people with employer-based insurance (ρ=–0.0114) in the low-risk bins. Individuals with high income, high education, and private insurance accounted for 26.62% (173/650), 14.00% (91/650), and 41.38% (269/650), respectively, in the high-risk and very high-risk categories.

## Discussion

### Summary of Main Findings

In this study, we developed a case-finding tool to identify general population individuals at risk of future cardiac dysrhythmia events within 1 year using Maine statewide health information exchange aggregated EHR data sets. The predictive model was trained retrospectively (AUROC 0.854) and validated prospectively (AUROC 0.827). Our model was capable of prospectively stratifying the general population into 5 risk bins (very low, low, medium, high, and very high) of incident arrhythmia. It will support targeted care plans to manage patients in different risk categories.

### Comparison With Prior Work

Our case-finding tool is different from previous efforts in terms of the targeted population and predictive timeframe. Other models [29,30] applied the logistic regression method, focusing on individuals with symptoms of syncope; the AUROC values of these models ranged from 0.44-0.81. Given that our method is be applicable to the general population, with a 1-year prospective timeframe, our case-finding tool has additional translational advantages.

### Model Risk Predictors and Their Implications for Preventive Care and Early Intervention

**Figure 7:**
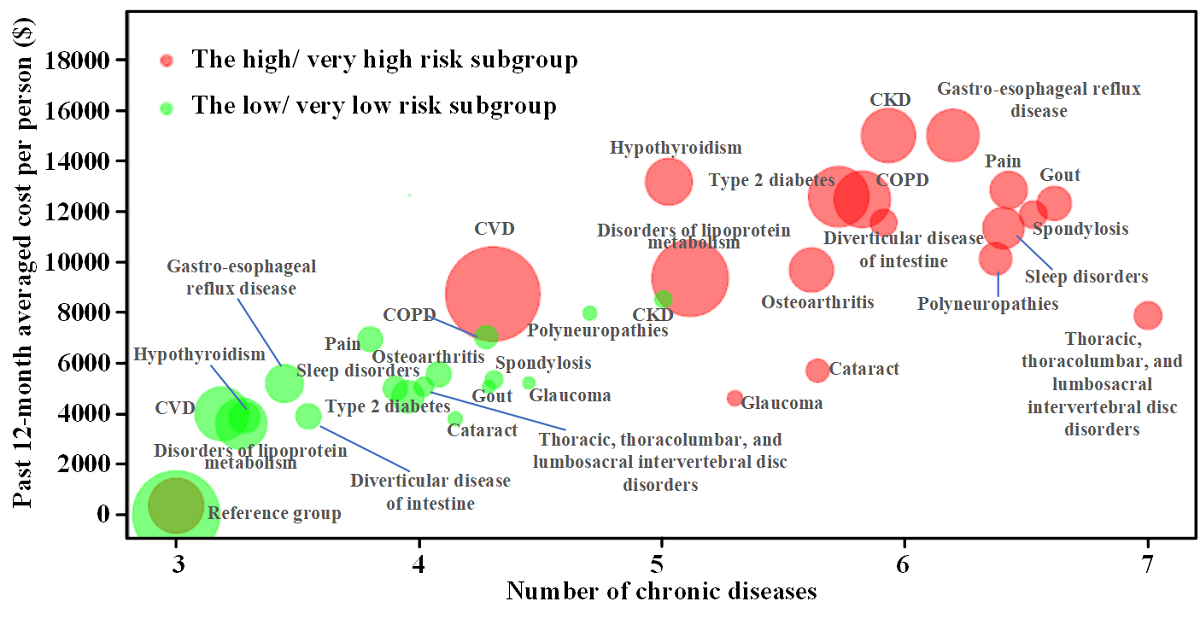
Patients' average clinical costs in the past 12 months with respect to the average number of chronic diseases; 17 common diseases are presented in the low- and very low–risk group (green) and the high- and very high–risk group (red). CKD: chronic kidney disease; COPD: chronic obstructive pulmonary disease; CVD: cardiovascular disease.

The high-risk or very high-risk bin individuals in a prospective cohort are likely to have higher disease burdens, given the confirmative diagnosis of multiple chronic diseases or acute disease events as well as other major medical histories (Figure 7 and Multimedia Appendix 11). Cardiovascular disease, one of the top predictors of our model, was found to be associated with heart failure, cardiomyopathy, and some valve problems [7]. Another important predictor of the model—chronic kidney disease—was also related to a few acute or chronic diseases that caused severe outcomes. A bidirectional causative relationship may exist between atrial ﬁbrillation and chronic kidney disease [31], and coexistence of atrial ﬁbrillation and chronic kidney disease greatly increased morbidity and mortality. Patients with chronic kidney disease may have a higher risk of death when implantable cardioverter defibrillators are used to treat ventricular arrhythmias [32]. Studies have shown that conditions with asymptomatic and persistent hypoglycemia increased the risk of arrhythmia [33]. Therefore, patients with chronic diabetes (including Type 1 and 2) often have varying degrees of arrhythmia risk. The direct effect of low glucose levels, hypokalemia, and catecholamine release can prolong cardiac repolarization, increasing the risk of early afterdepolarization and ventricular arrhythmias [33].

Studies have shown that certain social factors were indirect causes of arrhythmia. The predisposing factors of arrhythmia may involve various aspects of the psychosocial environment related to social status [34]. Social and psychosocial factors may influence the risk of arrhythmia through behavioral risk factors for smoking, exercise, and diet [35]. According to a survey study [36], the level of education and social status (occupational and occupational income) were inversely related to the incidence of cardiovascular disease. These observations are in line with the findings for socioeconomic factors included in our model: education, income, insurance type, regional features (towns, villages), and ethnic groups. We found that individuals with low incomes, low education levels, and nonprivate insurance groups had a higher probability of having arrhythmias (Multimedia Appendix 10).

### Practical Application of the Risk Model

Our study established a model for predicting the probability of arrhythmia disease within 1 subsequent year. By continuously tracking the influencing factors, the accuracy and applicability of the prediction results could be further improved. The predictive weight of different kinds of factors can provide insight into the formation mechanism of arrhythmia, the analysis of predisposing factors, and the research of preventive measures.

Our model will benefit physicians and health care organizations as well as patients. Model prediction and risk score results can be used as an auxiliary tool for physicians to diagnose and provide a reference for treatment planning. The stratification of the risk of the patient population also contributes to medical budget planning and target intervention. In the very high-risk category, 29.63% patients (8/27) would have been diagnosed with arrhythmia within the first 4 months of the subsequent year, which would then have gradually increased to 51.85% (14/27) within 1 year. Therefore, for those in the very high-risk group, it is necessary to formulate appropriate personalized intervention programs according to medical history, health status, living environment, and other conditions to prevent or delay the development of arrhythmia.

Given that our study showed that individuals at high risk of developing arrhythmias often have multiple chronic conditions, such as cardiovascular disease, metabolism disorders, type 2 diabetes, chronic obstructive pulmonary disease, and chronic kidney disease, aggressive interventions, including early routine testing and treatment of related chronic diseases, are needed for those patients. Arrhythmias after surgery are common and can lead to serious complications [37]. Therefore, for patients undergoing surgeries, especially chest and heart surgeries, it is necessary to take some preventive measures and conduct continuous electrocardiogram monitoring. This measure helps identify this high-risk population and avoids an increased risk of cardiovascular events and death [37]. Our model found that people with employer insurance, higher education, or higher incomes have a lower probability of experiencing arrhythmia. We speculate that these people tend to pay more attention to their physical condition and exercise more often to maintain health. Targeted early intervention reduces the number of arrhythmia patients and arrhythmia conditions, which is a rational and effective allocation of health care resources.

### Limitations

Our research has some limitations that could be further improved. First, some information was missing from our data set. Lifestyle information (such as eating habits and amount of daily exercise) was not fully documented in the EHR data. Second, when there are too many missing variables, there may be some bias in data preprocessing with the k-nearest neighbor method, resulting in inaccurate estimation results. Third, arrhythmia is a very common symptom that can be triggered in many cases, and some occurrences are not dangerous. Although arrhythmia was defined in detail in our model, it was only stratified by the probability of its occurrence, not by its severity. If the patient population based on the severity of the arrhythmia can be further subdivided, more accurate reference information for arrhythmia diagnosis and intervention will be provided.

### Conclusions

A risk prediction model of 1-year incidence of cardiac dysrhythmia was developed and prospectively validated using EHR data from 1.5 million people in Maine. The model was able to classify patients according to the predicted scores. The model had a good predictive performance (AUROC 0.827) in a prospective test. Age, gender, cardiovascular disease, chronic kidney disease, chest pain, pleural effusion, and socioeconomic factors were found to be related to new arrhythmia. For patients at high risk, early intervention should be carried out in a timely manner. Patients with low and moderate risk should maintain good living and eating habits, pay more attention to their physical condition, and exercise more often to avoid the occurrence of serious arrhythmias. This prediction model and analysis will ultimately benefit patient families, clinicians, and social health care institutions.

## Appendix

Multimedia Appendix 1 List of social determinant variables detailed in the data source and mapping method.

Multimedia Appendix 2 List of the top 60 important features and their odds ratios in the model.

Multimedia Appendix 3 Receiver operating characteristic curve comparisons of the model performance.

Multimedia Appendix 4 Receiver operating characteristic curves derived from the prospective cohorts.

Multimedia Appendix 5 Performance of the 1-year arrhythmia risk prediction model in the prospective cohort.

Multimedia Appendix 6 Distribution of age and gender in the 5 risk categories.

Multimedia Appendix 7 Distribution of top risk predictors across the 5 risk categories.

Multimedia Appendix 8 Time-to-arrhythmia diagnosis curves of the chronic disease group in the low-risk/very low-risk and high-risk/very high-risk populations of the prospective cohort.

Multimedia Appendix 9 (a) The feature importance score bar chart of health status, laboratory test, and medication. (b) Distributions of health status, laboratory test, and medication in the low-risk/very low-risk and high-risk/very high-risk categories.

Multimedia Appendix 10 Spearman rank correlation between socioeconomic features and prospective arrhythmia risk score.

Multimedia Appendix 11 Patients' average clinical costs and average number of chronic diseases in the past 12 months in the low-risk/very low-risk and the high-risk/very high-risk subgroups. The points presented 17 common diseases.

## Abbreviations

AUROC: area under the receiver operating characteristic curve
EHR: electronic health record
ICD-10-CM: International Classification of Diseases, Tenth Revision, Clinical Modification
LASSO: least absolute shrinkage and selection operator
PPV: positive predictive value
XGBoost: extreme gradient boosting
