# Supplementary material for: Electronic Health Record–Based Prediction of 1-Year Risk of Incident Cardiac Dysrhythmia: Prospective Case-Finding Algorithm Development and Validation Study
Source: JMIR Med Inform. 2021 Feb 17;9(2):e23606. doi: 10.2196/23606 (PMC7929752; doi:10.2196/23606)
Supplement: Multimedia Appendix 1 [file medinform_v9i2e23606_app1.docx]

**Appendix 1.** List of social determinant variables detailed in the data source and mapping method，collected from the US census and United States Department of Agriculture.

|  | **Social Economic Determinants** | **Levels** | **Variable Type** | **Mapping Method** | **Data Source** |
| --- | --- | --- | --- | --- | --- |
| 1 | Racial Demographics | Four levels: White; Black/African American; American Indian; Asian | % | ZIP code | US Census ACS (American Community Survey) |
| 2 | Urban or Rural | Two levels: Urban ratio; Rural ratio | % | ZIP code |  |
| 3 | Nativity and Citizenship |  | % | ZIP code |  |
| 4 | Education level | Four levels: Less than High school; High School; College associate bachelor; Graduate degree. | % | ZIP code |  |
| 5 | English proficiency |  | % | ZIP code |  |
| 6 | Health insurance status | Six levels: Public; Medicare; Medicaid; VA health care. Private: Employ based; Direct purchase; Tricare/military | % | ZIP code |  |
| 7 | Poverty or Household income |  | % | ZIP code |  |
| 8 | Unemployment rate |  | % | ZIP code |  |
| 9 | Percentage of population living within half mile to park |  | % | County level | Centers for Disease Control and Prevention, Community Health Status Indicators (CDC CHSI) |
| 10 | GINI inequality index |  | [0,1] | ZIP code | US Census (ACS) |
| 11 | Social Vulnerability Index |  | [0,1] | County level | CDC or Agency for Toxic Substances and Disease Registry (ATSDR) |
