# Supplementary material for: Electronic Health Record–Based Prediction of 1-Year Risk of Incident Cardiac Dysrhythmia: Prospective Case-Finding Algorithm Development and Validation Study
Source: JMIR Med Inform. 2021 Feb 17;9(2):e23606. doi: 10.2196/23606 (PMC7929752; doi:10.2196/23606)
Supplement: Multimedia Appendix 2 [file medinform_v9i2e23606_app2.docx]

**Appendix 2.** List of the top 60 important features and their odds ratios in the model.

| **Characteristic** | **Prospective cohort** | **Case (N=16,576)** | **n%** | **Control (N=1,024,191)** | **n%** | **Odds ratio** |
| --- | --- | --- | --- | --- | --- | --- |
| **Age(years)** |  |  |  |  |  |  |
| <35 | 399,545 | 1,021 | 6.2 | 398,524 | 38.9 | 0.32 |
| 35-50 | 176,995 | 1,298 | 7.8 | 175,697 | 17.2 | 0.1 |
| 50-65 | 243,161 | 4,033 | 24.3 | 239,128 | 23.3 | 0.01 |
| 65-75 | 135,600 | 4,573 | 27.6 | 131,027 | 12.8 | 0.15 |
| >75 | 85,466 | 5,651 | 34.1 | 79,815 | 7.8 | 0.27 |
| **Gender** |  |  |  |  |  |  |
| Female | 571,821 | 8,242 | 49.7 | 563,579 | 55.0 | 0.05 |
| Male | 468,946 | 8,334 | 50.3 | 460,612 | 45.0 | 0.05 |
| **Chronic disease** |  |  |  |  |  |  |
| Essential (primary) hypertension (CVD) | 218,851 | 8,270 | 49.9 | 210,581 | 20.6 | 0.3 |
| Chronic ischemic heart disease (CVD) | 45,327 | 3,052 | 18.4 | 42,275 | 4.1 | 0.14 |
| Nonrheumatic mitral valve disorders (CVD) | 10,203 | 794 | 4.8 | 9,409 | 0.9 | 0.05 |
| Nonrheumatic aortic valve disorders (CVD) | 9,787 | 884 | 5.3 | 8,903 | 0.9 | 0.04 |
| Heart failure (CVD) | 13,206 | 13,206 | 79.7 | - | - | 0.08 |
| Atrioventricular and left bundle-branch block (CVD) | 4,920 | 496 | 3 | 4,424 | 0.4 | 0.03 |
| Disorders of lipoprotein metabolism and other lipidemias | 215,074 | 7,492 | 45.2 | 207,582 | 20.3 | 0.25 |
| Gastro-esophageal reflux disease | 114,922 | 3,655 | 22 | 111,267 | 10.9 | 0.11 |
| Chronic obstructive pulmonary disease | 42,778 | 2,291 | 13.8 | 40,487 | 4 | 0.09 |
| Type 2 diabetes mellitus | 84,649 | 3,590 | 21.7 | 81,059 | 7.9 | 0.14 |
| Cardiomyopathy | 4,951 | 485 | 2.9 | 4,466 | 0.4 | 0.03 |
| Diverticular disease of intestine | 48,702 | 1,650 | 10 | 47,052 | 4.6 | 0.04 |
| Sleep disorders | 45,370 | 1,713 | 10.3 | 43,657 | 4.3 | <0.01 |
| Hypothyroidism | 74,495 | 2,301 | 13.9 | 72,194 | 7 | <0.01 |
| Chronic kidney disease | 23,653 | 1,586 | 9.6 | 22,067 | 2.2 | 0.05 |
| Complications and ill-defined descriptions of heart disease | 11,453 | 898 | 5.4 | 10,555 | 1 | 0.05 |
| **Acute disease** |  |  |  |  |  |  |
| Palpitations | 11,352 | 700 | 4.2 | 10,652 | 1 | 0.05 |
| Pain in throat and chest | 55,368 | 1,703 | 10.3 | 53,665 | 5.2 | 0.04 |
| Syncope and collapse | 11,855 | 474 | 2.9 | 11,381 | 1.1 | 0.06 |
| Edema | 17,768 | 1,264 | 7.6 | 16,504 | 1.6 | 0.03 |
| Abnormalities of breathing | 46,694 | 1,944 | 11.7 | 44,750 | 4.4 | <0.01 |
| Elevated blood glucose level | 28,287 | 841 | 5.1 | 27,446 | 2.7 | 0.03 |
| Aphagia and dysphagia | 12,693 | 489 | 3 | 12,204 | 1.2 | 0.02 |
| Malaise and fatigue | 46,834 | 1,531 | 9.2 | 45,303 | 4.4 | 0.04 |
| Dizziness and giddiness | 23,559 | 882 | 5.3 | 22,677 | 2.2 | 0.07 |
| Shortness of breath | 10,010 | 539 | 3.3 | 9,471 | 0.9 | 0.06 |
| Disorders of urinary system | 25,449 | 794 | 4.8 | 24,655 | 2.4 | 0.03 |
| **Health status** |  |  |  |  |  |  |
| Body mass index (BMI) >33.0 | 27,274 | 862 | 5.2 | 26,412 | 2.6 | 0.04 |
| Long term (current) drug therapy | 107,834 | 4,340 | 26.2 | 103,494 | 10.1 | 0.55 |
| Personal history of certain other diseases | 40,717 | 1,706 | 10.3 | 39,011 | 3.8 | 0.14 |
| Personal history of other diseases and conditions | 104,777 | 3,640 | 22 | 101,137 | 9.9 | 0.22 |
| Encounter for supervision of normal pregnancy | 11,045 | 49 | 0.3 | 10,996 | 1.1 | 0.05 |
| Presence of cardiac and vascular implants and grafts | 14,625 | 1,237 | 7.5 | 13,388 | 1.3 | 0.14 |
| Acquired absence of organs, not elsewhere classified | 20,430 | 714 | 4.3 | 19,716 | 1.9 | 0.03 |
| Presence of other functional implants | 21,992 | 968 | 5.8 | 21,024 | 2.1 | 0.06 |
| Other postprocedural states | 33,231 | 1,128 | 6.8 | 32,103 | 3.1 | 0.05 |
| **Lab test** |  |  |  |  |  |  |
| INR in Blood by Coagulation assay | 9,616 | 582 | 3.5 | 9,034 | 0.9 | 0.21 |
| Glomerular filtration rate/1.73 sq M.predicted [Volume Rate/Area] in Serum or Plasma by Creatinine-based formula (MDRD) | 16,726 | 772 | 4.7 | 15,954 | 1.6 | 0.18 |
| COHgb MFr Bld | 1,368 | 62 | 0.4 | 1,306 | 0.1 | <0.01 |
| Troponin T.cardiac [Mass/volume] in Serum or Plasma | 2,549 | 189 | 1.1 | 2,360 | 0.2 | 0.02 |
| Glucose | 1,797 | 90 | 0.5 | 1,707 | 0.2 | 0.46 |
| Creatine kinase Ser/Plas CCnc Pt Qn | 5,170 | 244 | 1.5 | 4,926 | 0.5 | 0.02 |
| Reticulocytes Bld NCnc Pt Qn | 1,802 | 92 | 0.6 | 1,710 | 0.2 | 0.01 |
| Natriuretic peptide.B prohormone [Mass/volume] in Serum or Plasma | 2,889 | 237 | 1.4 | 2,652 | 0.3 | 0.02 |
| **Procedure** |  |  |  |  |  |  |
| Gastrointestinal System, Excision | 13,407 | 369 | 2.2 | 13,038 | 1.3 | <0.01 |
| Heart and Great Vessels, Dilation | 2,223 | 143 | 0.9 | 2,080 | 0.2 | <0.01 |
| Subcutaneous Tissue and Fascia, Insertion | 567 | 50 | 0.3 | 517 | 0.1 | 0.01 |
| **Medication** |  |  |  |  |  |  |
| beta-Adrenergic Blocker | 69,572 | 4,454 | 26.9 | 65,118 | 6.4 | 1.47 |
| HMG-CoA Reductase Inhibitor | 98,358 | 5,054 | 30.5 | 93,304 | 9.1 | 1.29 |
| Loop Diuretic | 18,829 | 1,611 | 9.7 | 17,218 | 1.7 | 0.69 |
| Calcium Channel Blocker | 5,805 | 540 | 3.3 | 5,265 | 0.5 | 0.24 |
| Proton Pump Inhibitor | 69,278 | 2,928 | 17.7 | 66,350 | 6.5 | 0.89 |
| Vitamin K Antagonist | 5,541 | 642 | 3.9 | 4,899 | 0.5 | 0.38 |
| **Utilizations** |  |  |  |  |  |  |
| Mean of inpatient admission(s) | - | 0 | - | 0 | - | 0.11 |
| Mean of emergency visit(s) | - | 1 | - | 0 | - | 0.19 |
| Mean of outpatient visit(s) | - | 9 | - | 5 | - | 4.23 |
| Mean of inpatient day(s) | - | 1 | - | 1 | - | 0.61 |
| Medical cost(s) | - | 4,116 | - | 2,081 | - | 6.41 |
