# Supplementary material for: Electronic Health Record–Based Prediction of 1-Year Risk of Incident Cardiac Dysrhythmia: Prospective Case-Finding Algorithm Development and Validation Study
Source: JMIR Med Inform. 2021 Feb 17;9(2):e23606. doi: 10.2196/23606 (PMC7929752; doi:10.2196/23606)
Supplement: Multimedia Appendix 3 [file medinform_v9i2e23606_app3.docx]

**Appendix 3.** The ROC comparisons of the model performance. 1. Ensemble learning, 2. Lasso, 3. XGBoost, 4. Feed-forward Neural Network, 5. Boosting, 6. Random Forest, 7. KNN, 8. Naïve Bayes.
