# Supplementary material for: Electronic Health Record–Based Prediction of 1-Year Risk of Incident Cardiac Dysrhythmia: Prospective Case-Finding Algorithm Development and Validation Study
Source: JMIR Med Inform. 2021 Feb 17;9(2):e23606. doi: 10.2196/23606 (PMC7929752; doi:10.2196/23606)
Supplement: Multimedia Appendix 4 [file medinform_v9i2e23606_app4.docx]

**Appendix 4.** The ROC derived from the prospective cohorts.
