# Supplementary material for: Electronic Health Record–Based Prediction of 1-Year Risk of Incident Cardiac Dysrhythmia: Prospective Case-Finding Algorithm Development and Validation Study
Source: JMIR Med Inform. 2021 Feb 17;9(2):e23606. doi: 10.2196/23606 (PMC7929752; doi:10.2196/23606)
Supplement: Multimedia Appendix 5 [file medinform_v9i2e23606_app5.docx]

**Appendix 5.** The performance of the 1-year arrhythmia risk prediction model in the prospective cohort, summarized in PPV, sensitivity, and mean relative risk.

|  | **Very low** | **Low** | **Medium** | **High** | **Very high** | **Total** |
| --- | --- | --- | --- | --- | --- | --- |
| **Number of patients** | 555,233 | 466,594 | 18,290 | 623 | 27 | 1,040,767 |
| **True Positives** | 1,873 | 12,511 | 2,006 | 172 | 14 | 16,576 |
| **PPV (%)** | 0.3 | 2.7 | 11.0 | 27.6 | 51.9 | 1.6 |
| **Sensitivity (%)** | 11.3 | 75.5 | 12.1 | 1.0 | 0.1 | - |
| **Mean Relative Risk** | 0.2 | 1.7 | 6.9 | 17.3 | 32.6 | - |
