# Supplementary material for: Electronic Health Record–Based Prediction of 1-Year Risk of Incident Cardiac Dysrhythmia: Prospective Case-Finding Algorithm Development and Validation Study
Source: JMIR Med Inform. 2021 Feb 17;9(2):e23606. doi: 10.2196/23606 (PMC7929752; doi:10.2196/23606)
Supplement: Multimedia Appendix 7 [file medinform_v9i2e23606_app7.docx]

**Appendix 7.** Distribution of top risk predictors across the five risk categories.

| **Risk category** | **Very low** | **Low** | **Medium** | **High** | **Very high** |
| --- | --- | --- | --- | --- | --- |
| **Score intervals** | [0,1] | [1,10] | [10-30] | [30-45] | [45-55] |
| **Population** | 555233 | 466594 | 18290 | 623 | 27 |
| **Age(years)** |  |  |  |  |  |
| <35 | **68.5** | **4.2** | **0.6** | **0.6** | **0.0** |
| 35-50 | **22.8** | **10.7** | **1.4** | **0.6** | **0.0** |
| 50-65 | **7.4** | **42.9** | **9.5** | **6.3** | **0.0** |
| 65-75 | **0.9** | **26.9** | **27.6** | **25.4** | **7.4** |
| >75 | **0.4** | **15.3** | **60.9** | **67.1** | **92.6** |
| **Gender** |  |  |  |  |  |
| Female | **54.9** | **55.5** | **43.6** | **38.8** | **48.1** |
| Male | **45.1** | **44.5** | **56.4** | **61.2** | **51.9** |
| **Chronic disease** |  |  |  |  |  |
| CVD | **3.7** | **41.6** | **77.3** | **68.1** | **44.4** |
| Disorders of lipoprotein metabolism and other lipidemias | **4.7** | **39.4** | **60.0** | **47.7** | **25.9** |
| Gastro-esophageal reflux disease | **4.1** | **18.5** | **29.1** | **21.2** | **18.5** |
| Chronic obstructive pulmonary disease (COPD) | **0.6** | **7.6** | **22.7** | **24.6** | **22.2** |
| Type 2 diabetes mellitus | **1.5** | **15.1** | **30.1** | **28.6** | **18.5** |
| Diverticular disease of intestine | **0.8** | **9.1** | **9.9** | **5.3** | **3.7** |
| Sleep disorders | **1.6** | **7.3** | **12.7** | **13.2** | **7.4** |
| Hypothyroidism | **2.4** | **12.3** | **18.3** | **17.2** | **3.7** |
| Chronic kidney disease (CKD) | **0.3** | **4.0** | **18.6** | **22.5** | **22.2** |
| Pain in throat and chest | **1.9** | **7.4** | **10.6** | **10.6** | **7.4** |
| **Acute disease event** |  |  |  |  |  |
| Palpitations | **0.1** | **1.1** | **5.0** | **4.3** | **3.7** |
| Pain in throat and chest | **2.8** | **8.0** | **14.2** | **12.5** | **11.1** |
| Syncope and collapse | **0.6** | **1.6** | **4.8** | **4.2** | **7.4** |
| Edema | **0.5** | **2.8** | **10.5** | **13.6** | **7.4** |
| Abnormalities of breathing | **2.2** | **6.6** | **19.3** | **23.6** | **14.8** |
| Malaise and fatigue | **2.5** | **6.6** | **12.2** | **11.2** | **7.4** |
| Dizziness and giddiness | **1.1** | **3.4** | **7.7** | **7.2** | **7.4** |
| **Health status** |  |  |  |  |  |
| Body mass index (BMI) >33.0 | **1.3** | **3.5** | **4.5** | **3.9** | **3.7** |
| Long term (current) drug therapy | **3.4** | **17.5** | **38.0** | **40.8** | **22.2** |
| Personal history of other diseases and conditions | **5.2** | **20.3** | **38.6** | **36.0** | **25.9** |
| Presence of functional implants | **0.7** | **5.8** | **21.6** | **22.8** | **14.8** |
| Other postprocedural states | **1.5** | **5.0** | **8.2** | **8.3** | **11.1** |
| **Lab test** |  |  |  |  |  |
| INR in blood by coagulation assay | **0.5** | **1.4** | **5.3** | **12.7** | **22.2** |
| Glomerular filtration rate/1.73 sq M.predicted [Volume Rate/Area] in serum or plasma by creatinine-based formula (MDRD) | **0.3** | **2.6** | **7.2** | **9.3** | **3.7** |
| COHgb MFr Bld | **0.1** | **0.2** | **0.7** | **2.4** | **0.0** |
| Troponin T.cardiac [Mass/volume] in serum or plasma | **0.1** | **0.4** | **1.8** | **4.7** | **0.0** |
| Glucose | **0.1** | **0.3** | **0.8** | **1.1** | **0.0** |
| **Procedure** |  |  |  |  |  |
| Gastrointestinal system, excision | **0.2** | **1.2** | **1.1** | **0.6** | **0.0** |
| Heart and great vessels, dilation | **0.0** | **0.2** | **0.2** | **0.2** | **0.0** |
| Subcutaneous tissue and fascia, insertion | **0.0** | **0.1** | **0.3** | **1.0** | **0.0** |
| **Medication** |  |  |  |  |  |
| Beta-adrenergic blocker | **0.9** | **11.6** | **55.9** | **71.4** | **59.3** |
| HMG-CoA reductase inhibitor | **1.2** | **17.5** | **51.7** | **60.4** | **55.6** |
| Loop diuretic | **0.3** | **2.6** | **25.0** | **51.7** | **51.9** |
| Calcium channel blocker | **0.1** | **0.9** | **6.8** | **13.5** | **48.1** |
| Proton pump inhibitor | **2.2** | **11.2** | **27.0** | **25.5** | **25.9** |
| Vitamin K antagonist | **0.2** | **0.7** | **6.0** | **31.6** | **44.4** |
| **Utilizations** |  |  |  |  |  |
| Inpatient admission(s)> 0 | **5.9** | **7.8** | **17.1** | **19.4** | **25.9** |
| Emergency visit(s)> 0 | **28.6** | **25.6** | **37.0** | **40.0** | **25.9** |
| Outpatient visit(s)> 0 | **89.0** | **97.2** | **99.3** | **99.2** | **100.0** |
| Inpatient day(s)> 0 | **40.6** | **7.4** | **16.6** | **18.5** | **25.9** |
| Medical cost(s)> 2114 (Average cost) | **14.0** | **28.9** | **51.8** | **51.5** | **37.0** |
