# Supplementary material for: Electronic Health Record–Based Prediction of 1-Year Risk of Incident Cardiac Dysrhythmia: Prospective Case-Finding Algorithm Development and Validation Study
Source: JMIR Med Inform. 2021 Feb 17;9(2):e23606. doi: 10.2196/23606 (PMC7929752; doi:10.2196/23606)
Supplement: Multimedia Appendix 8 [file medinform_v9i2e23606_app8.docx]

**Appendix 8.** Time-to-arrhythmia diagnosis curves of the chronic disease subgroup in the low/ very low and high/ very high-risk population of prospective cohort.
