# Supplementary material for: Electronic Health Record–Based Prediction of 1-Year Risk of Incident Cardiac Dysrhythmia: Prospective Case-Finding Algorithm Development and Validation Study
Source: JMIR Med Inform. 2021 Feb 17;9(2):e23606. doi: 10.2196/23606 (PMC7929752; doi:10.2196/23606)
Supplement: Multimedia Appendix 9 [file medinform_v9i2e23606_app9.docx]

**Appendix 9(a).** The feature importance score bar chart of health status, laboratory test, and medication.


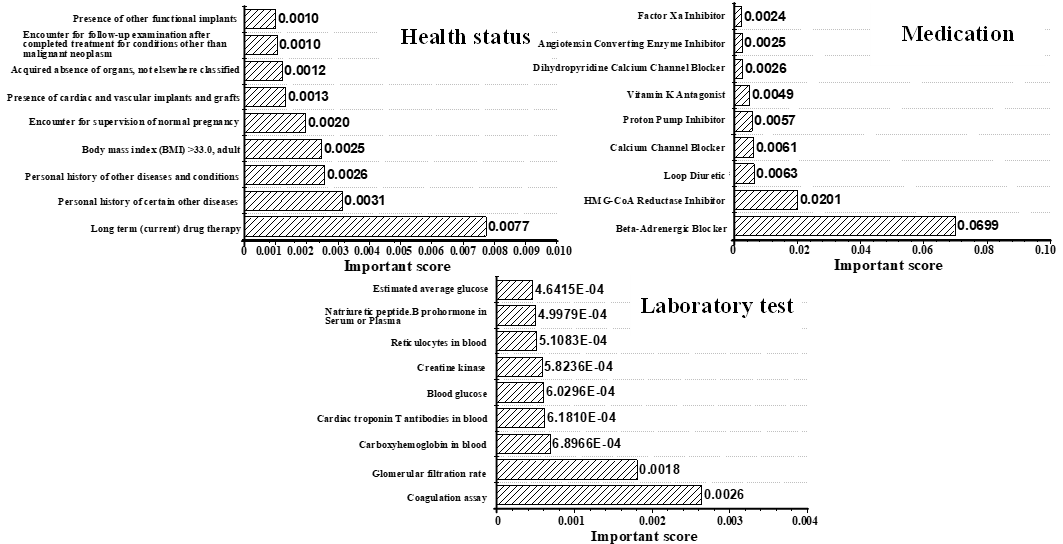


**Appendix 9(b).** Distribution about health status, laboratory test, and medication in the low/very low and high/very high-risk categories.
