# Supplementary material for: Electronic Health Record–Based Prediction of 1-Year Risk of Incident Cardiac Dysrhythmia: Prospective Case-Finding Algorithm Development and Validation Study
Source: JMIR Med Inform. 2021 Feb 17;9(2):e23606. doi: 10.2196/23606 (PMC7929752; doi:10.2196/23606)
Supplement: Multimedia Appendix 11 [file medinform_v9i2e23606_app11.docx]

**Appendix 11.** Patients average clinical costs and average number of chronic diseases in the past 12 months in the low / very low and the high / very high-risk subgroups. The points presented 17 common diseases.
